# External validation of the MidiCAT variant of thrombography: Comparison with calibrated automated thrombography and study of the centrifugation scheme

**DOI:** 10.3389/fcvm.2022.998687

**Published:** 2022-10-21

**Authors:** Sebastien Charles, Denis Guyotat, Pierre Fontana, Bernard Tardy, Thomas Lecompte, Emilie Chalayer

**Affiliations:** ^1^INSERM UMR 1059, Equipe SAINBIOSE, Université Jean Monnet, Saint-Étienne, France; ^2^Centre d’Investigation Clinique CIC 1408, CHU Saint Etienne, Saint-Étienne, France; ^3^Département d’Hématologie et de Thérapie Cellulaire, Institut de Cancérologie Universitaire, CHU Saint Etienne, Saint-Étienne, France; ^4^Geneva Platelet Group, Faculty of Medicine, University of Geneva, Geneva, Switzerland; ^5^Division of Angiology and Haemostasis, Geneva University Hospital, Geneva, Switzerland

**Keywords:** thrombin generation assay, blood coagulation tests, centrifugation, laboratory/methods, thrombomodulin

## Abstract

**Introduction:**

To perform Calibrated Automated Thrombography (CAT), the use of reduced plasma volumes (referred to as “MidiCAT”) makes it possible to more efficiently use limited volumes of valuable biobanked plasma samples and decreases expenses for reagents. It is, however, unclear whether the MidiCAT procedure is suitable when thrombin generation (TG) is studied in the presence of added thrombomodulin (TG-TM). Moreover, a simplified centrifugation scheme would facilitate biobanking, if appropriate, for more sensitive coagulation studies. We aimed to compare the results of “MidiCAT” (halved plasma and reagent volumes) with those from regular CAT, in the absence or presence of TM, as well as to study the impact of a single-centrifugation scheme for plasma preparation before freezing.

**Materials and methods:**

Plasma samples were prepared from the citrated blood from 20 Geneva hospital diverse patients without gross coagulation abnormalities with a single- or double-centrifugation scheme. Samples were kept frozen at −80°C and thawed just before the TG assay in duplicate under two conditions: 1 pM tissue factor (TF) or 5 pM TF + TM.

**Results and discussion:**

(1) We externally validated “MidiCAT” and also extended the validation to TG-TM. Whatever the method (CAT or MidiCAT), intra-assay (assessed with duplicates) CV was below 6% (1 pM TF) or below 10% (5 pM TF + TM) for ETP. Agreement between the MidiCAT and CAT results was satisfactory; the p coefficients were above 0.95 for ETP and above 0.90 for most other parameters; biases for ETP were +10.0% (1 pM FT) and +13.5% (5 pM + TM). (2) The centrifugation scheme markedly affected the results obtained in the presence of TM, whereas the bias and limit of agreement (difference plots) were low for the no TM condition. The bias in the presence of TM was obvious, more marked with plasma samples sensitive to TM when double centrifuged: the lower the ETP-TM, the greater the relative difference between the ETP-TM of plasma samples prepared with just single centrifugation and the reference plasma samples. Thus, a single-centrifugation procedure, as is often used for plasma biobanking, is suitable for TG study only if it is not performed in the presence of TM.

## Introduction

Many researchers strive to obtain plasma samples that are suitable for clinical study of the biomarkers of the haemostatic system. Currently, the complicated regulatory administrative procedures, the ever-increasing financial costs, and the time required to include patients in prospective studies lead to delays in performing such studies. Biobanks are a crucial resource for medical research. They can give researchers access to large and varying ranges of laboratory data about a substantial number of patients. Samples in biobanks and data derived thereof could be used in cross-purpose studies involving separate research teams. However, for haemostasis tests, the use of such samples is not possible due to the specific and stringent pre-analysis requirements. Both standardization and simplification of pre-analysis procedures could facilitate biobanking, however, they should not come at the expense of the accuracy of the results.

To better predict thromboembolic events in patients with cancer, as well as prognosis and therapy response, haemostatic biomarkers are the subject of intense research. Many biobanks are specializing in the field of cancer. It thus seems very important to determine whether haemostasis tests can be performed with frozen plasma samples from cancer biobanks.

Many pre-analysis factors can influence coagulation assays, be they traditional or innovative ones, and for thrombin generation (TG) in plasma. The residual platelet count after centrifugation is regarded as one of the most important factors. The residual platelet count depends on both the applied centrifugal forces and the method used to harvest the plasma (1, 2). To eliminate as many platelets as possible, which may contribute to the variability in TG results, double centrifugation is the general rule (3–6). There have been attempts at standardization between laboratories (7), with much emphasis placed on the pre-analysis phase (8). However, some teams still work with single centrifugation, for example, those working in the fields of cirrhosis or multiple myeloma (9–12). It still seems uncertain whether a single-centrifugation scheme can be applied for tests such as the Calibrated Automated Thrombinography (CAT).

As regards tissue factor (TF) concentrations, 1 or 5 pM are frequently used. Regarding the assessment of the risk of thromboembolism, CAT performed in the presence of thrombomodulin (TM) is widely considered to be relevant (13, 14) since TM mobilizes the protein C system. This might be particularly relevant since poor sensitivity to activated protein C, often referred to as “acquired resistance” in contrast to V Leiden, has been widely considered an important mechanism in cancer-associated hypercoagulability. Indeed, it has now been well established that in complex acquired coagulation disorders, such as in cirrhosis or during the neonatal period, there are differences between both coagulation factors and natural anticoagulants, and normal adult plasma samples.

The limited availability of stored plasma is another limitation for laboratory investigations performed with biobanked plasma samples. Indeed, CAT testing needs more plasma than most other haemostasis tests (duplicates require 320 μL of plasma). The MidiCAT procedure could be a convenient way to analyse plasma samples with limited volumes of biobanked plasma (14).

To address these issues, we first compared MidiCAT with CAT using frozen (after two centrifugations) thawed plasma samples; second, we compared a single-centrifugation to a double-centrifugation scheme before freezing the plasma, examining TG with MidiCAT.

## Materials and methods

### Study design, setting, and patients

This study was conducted in a university tertiary hospital (in Geneva). In line with a previous study about CAT pre-analytic variables (4), we picked up at random 20 plasma samples collected during daily clinical practice with enough volume left, with a few exclusion criteria as follows: (1) greatly extended prothrombin time and activated partial thromboplastin time (aPTT), (2) being under 18 years of age, (3) known as using anticoagulants, or (4) the patient explicitly refusing to give their general consent. This study was considered as falling outside of the scope of the Swiss legislation regulating research on human subjects so the need for local ethics committee approval was waived.

### Collection and handling of samples

Blood samples were collected into vacuum tubes containing 0.109 mol/L trisodium citrate 1 vol:9 vol of blood, that is, the collection tubes generally used for coagulation tests at the hospital (Vacutainer, Becton Dickinson). Blood samples were centrifuged within an hour after arrival at the laboratory. The first centrifugation (2,500 × *g* for 10 min, the standard centrifugation scheme in the laboratory) was performed by haemostasis technicians as per daily practice procedures. They harvested 70% of the supernatant above the buffy coat while taking care not to disturb the buffy coat so that cell contamination would be limited. The quality-assurance policy of the laboratory includes regular checks, which consistently document platelet count <10 × 10^9^/L. Half of the plasma volume was aliquoted into capped plastic tubes (Fischer-Scientific type MBP3464). The second centrifugation of the remaining half was performed by research technicians (2,500 × *g* for 15 min) and the supernatant was aliquoted into the same tubes. Plasma samples were stored at −80°C until testing. Commercial liquid normal pooled plasma (NPP; Cryocheck^®^, Cryopep, Montpellier, France, batches A1255 and A1260; citrate levels equivalent to 109 mM according to the purchaser) was used as a control. The centrifuge (Universal 320, Hettich GmbH, Tuttingen, Germany) was operated at 20°C using a light brake (15). All laboratory tests were performed within a week of storage. After being thawed for 5 min at 37°C, samples were immediately analysed.

### Laboratory assays

Thrombin generation (TG) was studied using Calibrated Automated Thrombography (CAT–Diagnostica Stago, Asnières-sur-Seine, France) with Stago reagents using Fluoroscan Ascent Fluorometer (version 5.0, Thermolab Systems, Helsinki, Finland). Runs were performed at 37°C after a 10 min pre-heating in the fluorometer of the round-bottom 96-well plate (Immulon 2HB) loaded with plasma samples and with procoagulant phospholipids and TF (see below). Fluo-Buffer together with FluCa was then automatically dispensed leading to recalcification of citrated plasma so that the reaction could start. Fluo-Buffer is a Hepes solution (pH 7.35) with calcium chloride, whereas Fluo-Substrate contains a thrombin-specific fluorogenic substrate (Z-GGR-AMC) solubilized in DMSO. To prepare FluCa, Fluo-Substrate was added to the warmed Fluo-Buffer shortly before the experiment. FluCa was freshly prepared for each run. Each TG run was calibrated according to the fluorescence curve obtained from a sample of the same plasma supplemented with a Thrombin Calibrator, with the specified amount of thrombin–α_2_–macroglobulin complex, and FluCa. Inner-filter effect and substrate consumption were accounted for as well. Fluorescence was recorded every 15 s for 60 min. All samples were analysed in duplicate. Parameters of interest were derived from each TG curve (i.e., thrombogram) using the Thrombinoscope software, version 5.0 (Diagnostica Stago, Asnières-sur-Seine, France). All experiments were conducted with the same batches of reagents. TM (rabbit lung; BioMedica Diagnostics Inc., Stamford USA) was added to the TG mixture to assess the dynamic inhibitory protein C system. We used a TM concentration of 1.75 nM to induce a 50% decrease in ETP compared with values in the absence of TM with normal plasma.

First, we compared MidiCAT with the CAT method using frozen (after two centrifugations)–thawed plasma samples. Second, we compared the TG of plasma samples prepared with either a single-centrifugation scheme or a double-centrifugation scheme using MidiCAT.

For the CAT method acting as reference, experiments were performed at a total volume of 120 μL. To initiate TG, 20 μL of the reagent comprising procoagulant phospholipid vesicles only (MP-reagent) or recombinant TF and procoagulant phospholipid artificial vesicles (either PPP-low reagent 1 pM TF or PPP reagent 5 pM TF) were added to 80 μL of plasma into each well. TG was eventually triggered with automated dispensing of 20 μL FluCa reagent.

For the MidiCAT method (Bloemen et al. (16)), experiments were conducted in a reduced total volume of 60 μL. All volumes of the CAT method were halved. To modify the dispensing volume, we went to the folder: C: \Program Files (x86) \Thrombinoscope \Users \(username) \Setting, opened the file: default.set with notepad located the line “nDispenseVolume”, and entered the desired volume.

### Statistical analysis

Median values (interquartile ranges, IQR) and figures (percentages) were used for descriptive purposes. Correlations between the results of TG obtained with the two CAT methods and with the two centrifugation schemes were determined with Spearman’s coefficient (p). To further evaluate the differences and biases in results between the two procedures, we plotted the percentage difference between the two methods ((B-A)/A × 100) against the ETP obtained with the reference method (methods A and B: reference and alternative procedure, respectively). Coefficients of variation (CV) were calculated for inter-well imprecision (standard deviation/mean × 100). The graphs and analyses were done using R software (version 3.5.0).

## Results

To first compare MidiCAT and CAT and then compare the single-centrifugation and double-centrifugation schemes, plasma samples from 20 patients were analysed. In addition, two commercial normal plasma pools were studied both with CAT and MidiCAT. On the whole, 133 duplicate thrombograms were obtained under different conditions. One half of the patient samples was used for the first part of the study and the other for the second part.

The median age of patients was 46 years (range: 24–89). There were eight female patients. Prothrombin times were all within the normal range, and the median aPTT was 25.5 s (normal range: 23.6–32.5 s).

Regardless of the method used (CAT or MidiCAT), intra-assay (assessed with duplicates) CV was below 6% for ETP with 1 pM TF and below 10% with 5 pM TF in the presence of TM.

### Comparison of the calibrated automated thrombography and MidiCAT methods

**FIGURE 1:**
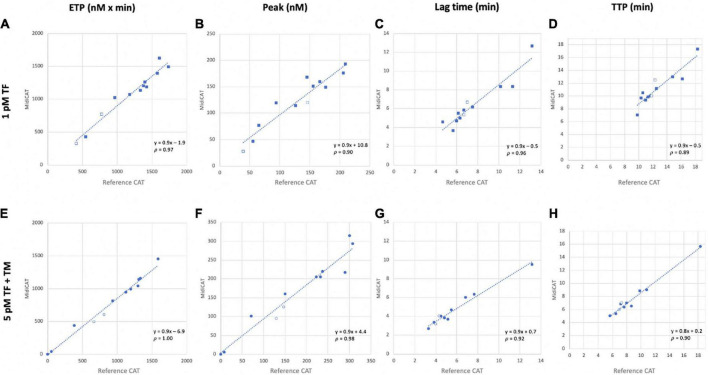
Correlation of thrombin generation parameters obtained with MidiCAT variant method with those of the reference method. Coagulation was initiated with 1 pM **(A–D)** and 5 pM TF in the presence of TM **(E–H)**. Plots for endogenous thrombin potential [ETP, nM × min; **(A, E)**], peak [nM; **(B, F)**], lagtime [min; **(C, G)**], and time to peak [TTP, min; **(D, H)**]. Filled dots: patients’ plasma samples; empty dots: commercial normal pooled plasma. Plasma’s volume used: 40 μL for MidiCAT method and 80 μL for CAT method. CAT, calibrated automated thrombography; TF, tissue factor; TM, thrombomodulin; p, Spearman’s rank correlation coefficient.

**FIGURE 2:**
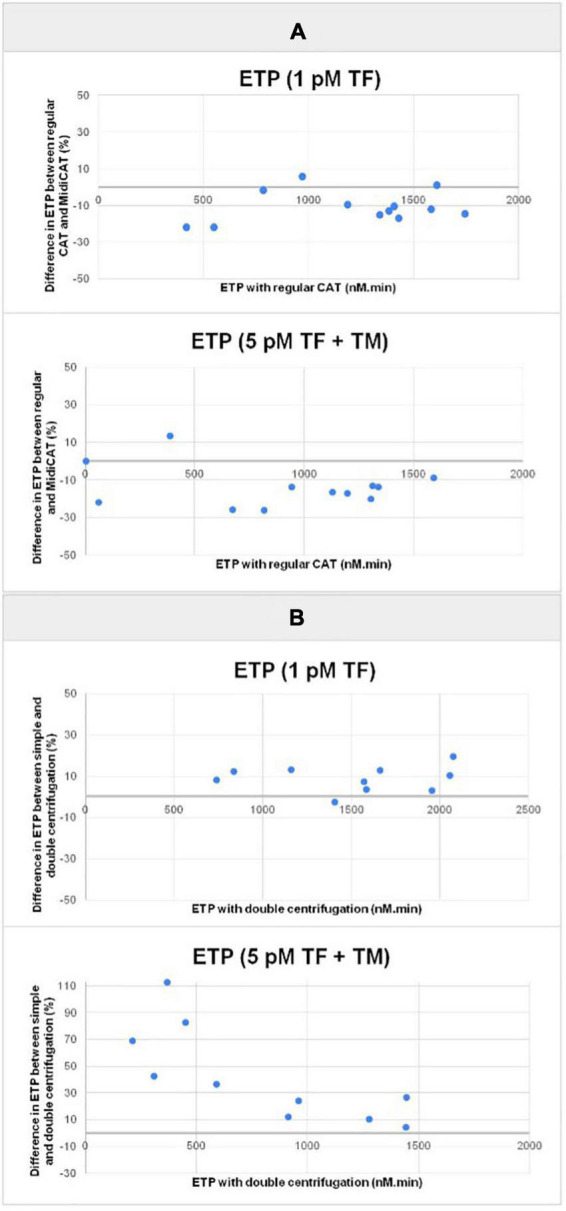
Plots of the difference in endogenous thrombin potential (ETP) between the variant methods and the reference method in percentage *vs.* endogenous thrombin potential (ETP) obtained with the reference method. TG was initiated with 1 pM TF alone, and with 5 pM TF combined with TM with 10 plasma samples (patients). Panel **(A)** comparison of MidiCAT variant method (40 μL plasma) with the reference method (CAT, 80 μL plasma)–plasma samples prepared with double centrifugation. Panel **(B)** comparison of single centrifugation and double centrifugation when preparing plasma; MidiCAT method. CAT, calibrated automated thrombography; ETP, endogenous thrombin potential; TM, thrombomodulin.

First, we compared MidiCAT with CAT using frozen (after two centrifugations)–thawed plasma samples under two experimental conditions: (1) low TF, to get the involvement of intrinsic tenase (anti-haemophilic factors) and (2) 5 pM TF in the presence of TM (at a concentration which halves the ETP of normal plasma), to investigate potential hypercoagulability. Numerical results are shown in Table 1. The correlation coefficients were above 0.95 for ETP (Table 1, Figure 1) and above 0.90 for the other parameters except for TTP with 1 pM of TF (Table 1). The biases for ETP (MidiCAT *vs*. CAT) were +10.0% (1 pM FT) and +13.5% (5 pM + TM) (Figure 2). ETP-TM of some plasma samples was high (hypercoagulable), as expected within the patient population. However, the ETP-TM of the other plasma samples would be undetectable, which was unexpected. This points to hypersensitivity to TM, the underlying reasons for which remain unknown.

**TABLE 1:** Median values and ranges of thrombin generation studies with the reference method and MidiCAT variant.

	Plasma preparation	
	
TG parameters	CAT 80 μL plasma	MidiCAT 40 μL plasma
**1 pM TF**		
ETP (nM × min)	1,359	1,160
	(417; 1,743)	(326; 1,626)
Peak (nM)	146	134
	(39; 209)	(27; 193)
Lag time (min)	6.7	5.7
	(4.7; 13.2)	(3.7; 12.7)
TTP (min)	11.8	10.3
	(9.8; 18.3)	(7.0; 17.3)
**5 pM TF + TM**		
ETP (nM × min)	1,035	879
	(0.0; 1,591)	(0.0; 1,452)
Peak (nM)	187	182
	(0.0; 314)	(0.0; 308)
Lag time (min)	4.7	3.9
	(2.7; 13.2)	(0.0; 9.5)
TTP (min)	7.5	6.7
	(5.0; 18.3)	(0.0; 15.7)

TG was initiated with 1 pM TF alone and with 5 pM TF combined with TM with 10 patients’ plasma samples. CAT, calibrated automated thrombography; ETP, endogenous thrombin potential; TTP, time to peak; TF, tissue factor; TG, thrombin generation; TM, thrombomodulin.

### Comparison of the two centrifugation schemes

As a general rule, plasma preparation consists of two sequential centrifugations. We compared a single-centrifugation scheme with the double-centrifugation scheme as a reference using the MidiCAT protocol to study TG. Numerical results are shown in Table 2. Correlation coefficients were above 0.95 for ETP (Table 2, Figure 2). Correlation coefficients were above 0.90 for peak and TTP. Bias and limit of agreement of the difference plots were low for the no TM value but in its presence, there was an obvious bias, which is more pronounced when plasma samples were sensitive to TM: the more sensitive the plasma, the greater the relative difference of ETP-TM of plasma samples prepared with just single centrifugation to the reference plasma with double centrifugation (Figure 2). Coefficients of variation were below 8% with regard to inter-well imprecision for ETP, peak, and TTP.

**TABLE 2:** Median values and ranges of thrombin generation studies–single *vs.* double centrifugation scheme to prepare plasma samples.

	Plasma preparation before freezing	
	
TG parameters	Single centrifugation	Double centrifugation
**1 pM TF**		
ETP (nM × min)	1,664	1,578
	(798; 2,486)	(738; 2,075)
Peak (nM)	258	239
	(89; 423)	(69; 333)
TTP (min)	6.8	7.2
	(5.0; 10.7)	(5.3; 11.7)
Lag time (min)	3.8	3.7
	(2.8; 10.7)	(2.7; 7.7)
**5 pM TF + TM**		
ETP (nM × min)	922	751
	(354; 1,829)	(210; 1,443)
Peak (nM)	207	168
	(62; 386)	(34; 299)
TTP (min)	6.8	7.8
	(5.0; 16.0)	(5.7; 25.3)
Lag time (min)	5.2	4.2
	(3.3; 23.3)	(3.0; 14)

TG was initiated with 1 pM TF alone and with 5 pM TF combined with TM with 10 plasma samples (patients). ETP, endogenous thrombin potential; TTP, time to peak; CAT, calibrated automated thrombography; TF, tissue factor; TG, thrombin generation; TM, thrombomodulin.

**FIGURE 3:**
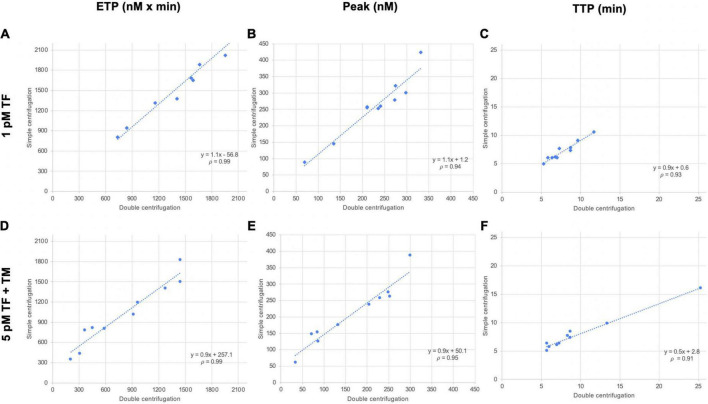
Correlation of thrombin generation parameters of plasma samples prepared with two centrifugation schemes. Ten plasma samples (from patients) were prepared with single centrifugation or double centrifugation, and TG was initiated with 1 pM TF and 5 pM TF in the presence of TM. Scattergrams of endogenous thrombin potential [ETP, nM × min; **(A, D)**], peak [nM; **(B, E)**], and time to peak [TTP, min; **(C, F)**]. TF, tissue factor; TM, thrombomodulin; p, Spearman’s rank correlation coefficient.

Additionally, contact phase activation in those plasma samples was estimated by studying TG without the addition of TF (MP-reagent: no TF; 4 μM phospholipids). When the results of this condition were compared with the ones where TF was added to get a final concentration of 5 pM, thrombograms were delayed and ETP was lower. The average differences in ETP were 39 and 77%, with single centrifugation or double centrifugation, respectively, indicating that the centrifugation scheme does indeed have a substantial effect on ETP. On average, ETP with no added TF was 60% greater in plasma samples prepared with a single centrifugation, than in those prepared with double centrifugation (Figure 3).

## Discussion

Calibrated Automated Thrombography (CAT) is the monitoring of thrombin generation and decay in clotting plasma, in short TG. CAT is widely used in the research field of haemostasis. Although, some current issues, such as sample volumes, limit the use of CAT. Experts have recommended performing CAT at the very least in duplicate (6). A calibrator must be run in parallel with the sample in which TG is measured. This determines the required volume of plasma. We have reported in this paper an external validation of “MidiCAT” (halved plasma and reagent volumes), as well as demonstrating its use for TG-TM. The TG tests were performed mostly with 5 pM TF. However, to sensitize CAT to hypercoagulable states, experiments should be conducted with the addition of TM. It allows for the assessment of the effect of the PC system on TG, according to the pioneering work of Dargaud (8) and its use afterward (14, 17–20). Not adding TM in CAT assays might explain why several studies have failed to show a significant correlation with the risk of venous thrombosis. More and more teams choose the experimental condition with added TM to investigate hypercoagulability. Thus, the necessary plasma volume for each CAT measurement (with and without TM) exceeds 480 μL. Bloemen et al. demonstrated that low plasma volume (40 μL) can be used with 5 pM TF, provided that all other volumes which are to be added to the reaction wells are halved as well (16). However, their measurements were performed with in-house reagents and only with 5 pM TF. Our results are, to date and to the best of our knowledge, the only reported demonstration whose results obtained from thrombograms using low plasma volume, that is, “MidiCAT”, do not deviate substantially from those obtained by CAT, the reference method. The results did indeed correlate well, when using the commercially available reagents (Stago; 5 pM TF + phospholipids–PPP Reagent, with added TM; and 1 pM TF + phospholipids–PPP Low Reagent). Spearman’s rank correlation coefficient for ETP is at least 0.97. Bloemen et al. achieved similar results with 5 pM and 0 pM procedures (16). The higher surface-to-volume ratio might explain, at least in part, the biases which were about +10% (MidiCAT *vs*. CAT). Moreover, it is important to note that costs are almost halved thanks to the decreased volumes of reactants.

There are many cancer biobanks, however, most of the time, the pre-analysis requirements before freezing for these biobanks do not correspond to pre-analysis requirements for haemostasis tests. We evaluated two centrifugation schemes to harvest plasma before freezing: the reference one, which consists of two sequential centrifugations (the goal is to leave as few platelets as possible, since they influence TG by providing procoagulant membranes and various internal proteins) (21); the simplified one, with just single centrifugation. Our data showed matching results between tested frozen plasma samples, after the two methods for all TG parameters obtained with MidiCAT when coagulation was initiated with 5 pM TF in the absence of TM, but not in the presence of the latter. These results are in line with those of Loeffen et al., who compared TG in plasma samples obtained from 12 healthy volunteers, centrifuged once at 2,000 × *g* for 15 min *vs.* 2,000 × *g* for 5 min followed by second centrifugation of harvested plasma at 10,000 × *g* for 10 min. No difference was reported when 5 pM TF was used to initiate TG (4). Loeffen et al., however, recommended a double-centrifugation scheme in presence of phospholipids to evaluate the level of contact activation on TG, probably because of their results with the low TF concentration (1 pM) or in the absence of added TF. Synthetic phospholipids contained in MP-reagent can be sufficient to enhance TG initiated by FXIIa in some patients (22). Surprisingly, in Loeffen’s study, plasma collected in vacutainer tubes with no added TF showed no significant differences in TG results in contrast to our results. However, a significant increase in ETP was found in the single-centrifugation samples compared to the double-centrifugation ones with 1 pM TF (23). On average, when no TF was added, we observed an increase in ETP between single-centrifuged and double-centrifuged plasma. These differences may be explained by the fact that the participants in our study were patients and not healthy volunteers. Moreover, our second centrifugation was at 2,000 × *g* for 15 min and not 10,000 × *g* for 10 min. It should be noted that some patient plasma samples might undergo a TF exposure phenomenon due to residual platelets (4). However, this seems to have had no impact on TG results initiated with 5 pM TF. Thus, based on our results, we suggest the possibility of using centrifuged frozen samples, routinely and primarily collected for other purposes, to perform TG with the MidiCAT method in the presence of phospholipids with initiation at 5 pM TF. This possibility is worth considering in future studies. Contact phase activation is a major problem at lower picomolar TF concentrations and this effect can be more pronounced when only a single centrifugation is performed, as we have found while adding only procoagulant phospholipids without TF (MP-reagent from Stago) (data not shown).

Regarding the results in the presence of TM, they are consistent with those of Lisman’s team (24) which showed that normally sensitive plasma may become resistant. What makes such plasma samples less sensitive to TM remains to be elucidated, but alterations to protein C and protein S are unlikely. Second centrifugation after plasma thawing has been proposed as an option, but it has not been investigated which undesirable component is removed in this way. It could be an option which was recently proposed (24). This remains to be independently confirmed.

We have to keep in mind that TG varies due to the conditions under which blood is collected, namely due to the tubes used and if the preparation/manipulation is carefully performed. Bias can be induced by contact activation, by residual platelets, and/or by impact quantification of the effects on analytical precision (4). Some researchers have estimated that not taking this into account may lead to an overestimation of TG (4). The use of CTI prevents contact phase activation. Although CTI has impracticalities and related costs, it can be omitted as long as TF is added to get a 5-pM concentration. We suggest that using frozen citrated plasma samples (without CTI) after single centrifugation is feasible. Of note, blood is often collected into vacuum tubes containing only citrate, as has been done in the current study. Results might differ when using other tubes, such as Monovette^®^ Sarstedt (4). Unfortunately, such specific conditions (tubes and preparation) are difficult to comply with and preclude the use of existing frozen plasma samples collected after just one centrifugation. Conducting a large prospective study with CAT is also difficult due to such stringent pre-analysis constraints and the associated therein costs. In the present study, we focused on suitable pre-analysis conditions that could permit turning regularly collected biobanked samples into usable material for CAT studies. Being able to use such conditions would permit for broader CAT applicability in line with the availability of the newly commercialized analyser ST-Genesia, notably for centrifugation schemes (25).

Contrary to the previous study on centrifugation schemes (4), ours was conducted with patient plasma samples and not samples from healthy volunteers. In addition, the centrifugation scheme was the one used in daily practice. Thus, our results are relevant to daily practice and readily applicable to clinical research. Our study has several limitations though. First, the diseases that the patients suffered from were unknown, and so abnormal TG phenotypes could not be explained. Of note, there was a large variation in TM reduction among patients’ plasma samples. Some plasma samples could have presented pre-activation for any reason or could have contained unnoticed anticoagulant drugs. Second, the double-centrifugation scheme that was used as comparison (2,500 × *g* for 10 min) is the mandated one in our institutions. On the other hand, some teams have used a 15 min centrifugation and/or second centrifugation at 10,000 × *g*, thus we cannot exclude that this might have affected the results. Third, only MidiCAT was tested for the comparison of the centrifugation schemes. However, it is unlikely that different results have been obtained using these schemes with regular CAT. Eventually, TG measurement in presence of 5 pM TF without added TM was not included in our study. Other studies are needed to prove that the condition without the addition of TM can be used with plasma obtained after single centrifugation and with reduced volume, aka MidiCAT.

Whether the volume of plasma required for TG studies can be reduced is a significant issue of practical importance. We have provided the first external validation of the MidiCAT procedure and conclude that accurate measurement of TG curves is feasible with half of the volume of the reference CAT method. The second issue we addressed was the simplified but still acceptable pre-analysis conditions. With this study, we aim to raise awareness of the lack of impact of artifactual contact activation or residual platelets on TG when using reagents containing 5 pM TF. We believe that our results add sufficient data to the previous study to allow for the use of the MidiCAT method with 5 pM TF with TM or with 1 pM TF (now referred to as, respectively, intermediary and low picomolar concentrations of TF). Moreover, the use of single-centrifuged plasma samples from biobanks for MidiCAT with 5 pM TF seems acceptable, provided no TM is added. A double-centrifugation scheme is recommended otherwise.

## Data availability statement

The raw data supporting the conclusions of this article will be made available by the authors, without undue reservation.

## Ethics statement

The studies involving human participants were reviewed and approved by Commission Cantonale d’Ethique de Genève (hospital general consent for blood from required standard analyses when clinical data are not required). Written informed consent for participation was not required for this study in accordance with the national legislation and the institutional requirements.

## Author contributions

EC, SC, and TL designed the study, performed the analysis, and wrote the manuscript. SC and EC performed the experiments and collected the data. SC, DG, PF, and BT participated in data analysis. All authors contributed to the manuscript.
